# Towards personalized precision functional mapping in infancy

**DOI:** 10.1162/imag_a_00165

**Published:** 2024-05-10

**Authors:** Lucille A. Moore, Robert J. M. Hermosillo, Eric Feczko, Julia Moser, Sanju Koirala, Madeleine C. Allen, Claudia Buss, Greg Conan, Anthony C. Juliano, Mollie Marr, Oscar Miranda-Dominguez, Michael Mooney, Michael Myers, Jerod Rasmussen, Cynthia E. Rogers, Christopher D. Smyser, Kathy Snider, Chad Sylvester, Elina Thomas, Damien A. Fair, Alice M. Graham

**Affiliations:** Masonic Institute for the Developing Brain, University of Minnesota, Minneapolis, MN, United States; Department of Pediatrics, University of Minnesota, Minneapolis, MN, United States; Institute of Child Development, University of Minnesota, Minneapolis, MN, United States; Department of Psychiatry, Oregon Health & Science University, Portland, OR, United States; Institute of Medical Psychology, Charité-Universitätsmedizin Berlin, Corporate Member of Freie Universität Berlin and Humboldt-Universität zu Berlin, Berlin, Germany; Department of Pediatrics, School of Medicine, University of California, Irvine, Irvine, CA, United States; Department of Psychiatry, University of Vermont, Burlington, VT, United States; Department of Behavioral Neuroscience, Oregon Health and Science University, Portland, OR, United States; Minnesota Supercomputing Institute, University of Minnesota, Minneapolis, MN, United States; Department of Medical Informatics & Clinical Epidemiology, Oregon Health & Science University, Portland, OR, United States; Department of Psychiatry, Washington University, St. Louis, MO, United States; Department of Pediatrics, University of California, Irvine, CA, United States; Departments of Neurology, Radiology, and Pediatrics, Washington University School of Medicine, St. Louis, MO, United States; Department of Neuroscience, Earlham College, Richmond, IN, United States; College of Education and Human Development, University of Minnesota, Minneapolis, MN, United States

**Keywords:** precision network mapping, resting-state fMRI, resting-state functional brain networks, infants, brain development, template matching

## Abstract

The precise network topology of functional brain systems is highly specific to individuals and undergoes dramatic changes during critical periods of development. Large amounts of high-quality resting state data are required to investigate these individual differences, but are difficult to obtain in early infancy. Using the template matching method, we generated a set of infant network templates to use as priors for individualized functional resting-state network mapping in two independent neonatal datasets with extended acquisition of resting-state functional MRI (fMRI) data. We show that template matching detects all major adult resting-state networks in individual infants and that the topology of these resting-state network maps is individual-specific. Interestingly, there was no plateau in within-subject network map similarity with up to 25 minutes of resting-state data, suggesting that the amount and/or quality of infant data required to achieve stable or high-precision network maps is higher than adults. These findings are a critical step towards personalized precision functional brain mapping in infants, which opens new avenues for clinical applicability of resting-state fMRI and potential for robust prediction of how early functional connectivity patterns relate to subsequent behavioral phenotypes and health outcomes.

## 1 Introduction

Over the past two decades, research has shown that cognitive functions arise from interactions among distributed brain regions within large-scale networks (Byington et al., 2023; Raichle, 2011). This shift from mapping functions to isolated regions of the brain has led to re-examination of variations in brain function and organization across individuals (Miranda-Dominguez et al., 2014; Seitzman et al., 2019), mental health conditions (Berman et al., 2014; Feczko et al., 2019), and development (Grayson & Fair, 2017; Marek et al., 2019; Moore et al., 2023). Recent evidence shows that characterization of brain-behavior relationships may be limited by the inherently low signal-to-noise ratio of functional neuroimaging data, the analysis of which not only requires thousands of subjects to be sufficiently powered, but also has effect sizes that are too small for precise functional characterization and clinical applicability (Gratton et al., 2022; Marek et al., 2022). Fortunately, these challenges can be overcome by precision functional mapping utilizing resting-state functional MRI (rs-fMRI) from highly sampled individuals.

Resting-state fMRI reveals that spontaneous, large-amplitude, low-frequency fluctuations in the blood-oxygen-level-dependent (BOLD) fMRI signal are temporally correlated across functionally related brain regions (Biswal et al., 1995; Gordon, Laumann, Gilmore, et al., 2017). Multiple studies leveraging graph theory and machine-learning algorithms have identified a fairly consistent set of 7–14 networks in adult rs-fMRI (Fair et al., 2008; Gordon et al., 2016; Miranda-Dominguez et al., 2018; Power et al., 2011; Smith et al., 2015; Yeo et al., 2011) that show striking correspondence with topological patterns of task-evoked activity (Biswal et al., 1995; Dosenbach et al., 2008; Gordon, Laumann, Gilmore, et al., 2017; Gratton et al., 2018; Vazquez-Trejo et al., 2022). Furthermore, differences in functional network strength or configuration have been described in a large variety of neurological disorders (Buckner et al., 2009; Cuthbert & Insel, 2013; Fair et al., 2010; Feczko et al., 2019; Hohenfeld et al., 2018; Insel et al., 2010; Y. Liu et al., 2008; Miranda-Domínguez et al., 2020; Segal et al., 2023; Silasi & Murphy, 2014; Supekar et al., 2008; L. Wang et al., 2009).

Resting-state fMRI is a particularly useful tool for examining functional brain organization in early infancy and across development because it can be conducted during natural sleep (Beck et al., 2011; Graham et al., 2015; Raichle, 2010; Zhang et al., 2019). Prior rs-fMRI research indicates that the foundational network architecture of the brain is already established both *in ute*ro (Doria et al., 2010; Moore et al., 2023; Thomason, 2020; Thomason et al., 2015; Turk et al., 2019) and in early infancy (De Asis-Cruz et al., 2015; Fransson et al., 2007, 2011; Gao, Alcauter, Smith, et al., 2015; Thomason et al., 2013). Various networks identified in infants nascently correspond with adult networks, with variation in ease of detection and developmental timelines (De Asis-Cruz et al., 2015; Gilmore et al., 2018; Grayson & Fair, 2017; Lin et al., 2008; Moore et al., 2023; Nielsen et al., 2022; van den Heuvel et al., 2015). Remarkably, these emerging networks exhibit similar organization and network selectivity to their adult counterparts in both short-range connectivity between anatomically proximal regions and long-range global connections between anatomically distant regions (Damaraju et al., 2014; Fransson et al., 2007; Gao et al., 2009; Moore et al., 2023; Smyser et al., 2010; Sylvester et al., 2022; Wylie et al., 2014).

Variability in the strength of connectivity of these emerging networks is associated with genetic and environmental factors and subsequent risk for neurodevelopmental disorders (Emerson et al., 2017; Graham et al., 2021; Luo et al., 2022; Rudolph et al., 2018; Van den Bergh et al., 2020; Wazana et al., 2021). However, it has been difficult to clinically leverage these findings towards, for example, identifying robust biomarkers for psychiatric conditions (Kraus et al., 2023) or developing precision medicine techniques to deliver highly neuroanatomically targeted clinical interventions (Gratton et al., 2018). One avenue to identify reliable cross-sectional brain-behavior associations in the general population is an epidemiological policy-based approach utilizing large-scale consortia studies (Gratton et al., 2022). However, these studies require thousands of participants in order to be sufficiently powered and ultimately explain relatively little individual-level variance in behavior (r ≤ 0.16), which may be useful for informing policy decisions, but not individual clinical care (Gratton et al., 2022; Marek et al., 2022).

An alternative to large-scale consortia studies is to use highly sampled individual subject data and increase effect sizes via highly focused study designs that maximize signal and minimize noise (Gordon, Laumann, Gilmore, et al., 2017; Gratton et al., 2022; Newbold et al., 2020). Within this category of study design is precision functional mapping, which presents a highly detailed picture of the functional architecture of the brain. The goal of precision functional mapping is to generate maximally precise or stable personalized brain maps, beyond which addition of more data does not significantly impact topological variation. Recent research has utilized precision mapping to pursue novel lines of inquiry, revealing remarkable neural plasticity in adulthood (Newbold et al., 2020) as well as distinct cortical network connectivity within subregions of multiple brain structures (Braga et al., 2019; Braga & Buckner, 2017; Reznik et al., 2023; Sylvester et al., 2020; Xue et al., 2021). Precision mapping is furthermore essential for thorough interrogation of individual differences. A growing body of research shows that the precise network topology of functional brain systems is highly specific to individuals across development, including in neonates (Molloy & Saygin, 2022), younger children and adolescents (Cui et al., 2020; Hermosillo et al., 2022), young adults (Gordon, Laumann, Adeyemo, et al., 2017), and adults (Dworetsky et al., 2021; Gordon, Laumann, Gilmore, et al., 2017; Gratton et al., 2018; Greene et al., 2020; Marek et al., 2018; Segal et al., 2023). Individual network distinctions are lost in most studies due to averaging (Gordon, Laumann, Adeyemo, et al., 2017; Laumann et al., 2015), obscuring important individual differences in neural organization and behavior (Marek et al., 2022; Reznik et al., 2023; Wolfers et al., 2018). In infants, precision mapping has potential to significantly advance robust, clinically applicable interpretation of early functional connectivity patterns for subsequent behavioral phenotypes and health outcomes (Gratton et al., 2022; Kraus et al., 2023).

Precision mapping in adults has involved extensive acquisition of rs-fMRI data across multiple sessions (Gordon, Laumann, Gilmore, et al., 2017; Gratton et al., 2018; Laumann et al., 2015; Newbold et al., 2020), followed by application of Infomap, a powerful data-driven community detection method, to reliably define individual network topology (Gates et al., 2016; Lancichinetti & Fortunato, 2009; Power et al., 2011; Rosvall & Bergstrom, 2008). For example, the Midnight Scan Club dataset acquired by Gordon, Laumann, Gilmore et al., (2017) includes an impressive 5 hours of rs-fMRI and 6 hours of task fMRI data acquired for 10 adults. Unfortunately, precision mapping has not yet been feasible in infants due in part to practical barriers in acquiring such large amounts of rs-fMRI data (see Dubois et al., 2021 and Korom et al., 2021 for an overview of practical challenges). Therefore, in infants, algorithms that require relatively modest amounts of rs-fMRI data to perform personalized precision functional network mapping are of critical importance.

Compared to data-driven community detection methods like Infomap, approaches that use group-average parcellations as priors require significantly less resting-state data (e.g., 10 vs. 40-60 minutes) (Gordon, Laumann, Gilmore, et al., 2017; Hermosillo et al., 2022; Laumann et al., 2015) and are thus well suited for the inherent challenges of infant data acquisition and quality. One such approach is template matching (TM), a method developed by Gordon, Laumann, Adeyemo, et al., (2017) for precision mapping in adults that uses group-average resting-state network templates as priors for robust and reliable identification of individual-specific network maps. Our group recently demonstrated that template matching could generate high-precision network maps in 9-10 year old adolescents with as little as 10 minutes of low-motion resting-state data and individualized maps with as little as 2 minutes (Hermosillo et al., 2022). This suggests that template matching could potentially be used for personalized precision functional mapping in infancy.

Template matching is well suited to accommodate unique characteristics of infant functional connectivity. Though various methods have been used to identify infant resting-state networks from group average data, few have generated individualized networks. Prior studies in infants (Eyre et al., 2021; Molloy & Saygin, 2022) as well as a portion of studies in older age groups (Kong et al., 2019; Yeo et al., 2011) employed clustering algorithms (e.g., k-means, independent component analysis, etc) to define group-level network parcellations which can then be regressed to individual subject data to generate individualized network maps. One issue is that the optimal number of clusters/components must be decided *a priori*, which can be difficult to determine and directly impacts how many networks are detected. In addition, network solutions are generated by comparing the strength of connection weights, which biases network detection towards strong, short-range connectivity. Selective long-range connectivity characteristic of adult functional networks is present already in the neonatal period, but is weaker and more difficult to detect even in group data, which has led to variability in robust network detection in the infant literature (Sylvester et al., 2022). Template matching relies on spatial patterns of connectivity and is thus less biased in this regard.

In the current study, we aimed to advance understanding of individual network topology to lay groundwork towards personalized precision functional mapping in infancy. Specifically, we sought to: (1) generate a set of infant-specific resting-state network templates; (2) employ these templates as priors for template matching to generate individualized resting-state network maps for two independent neonatal datasets (including comparison to Infomap-derived network maps); and (3) evaluate the reliability of template matching-derived individualized networks and the amount of data needed to achieve high precision. This work seeks to set the stage for reliable identification of individualized resting-state network topologies in infants, a critical step to advance our understanding of how early brain development relates to emergence of subsequent neurodevelopmental and psychiatric disorders.

## 2 Methods

### 2.1 Subject Information

Written informed consent was obtained from all parents of neonatal and adolescent participants, and additional assent was obtained from adolescent participants. We used rs-fMRI data from a cohort of 69 neonates from University of California Irvine (UCI) to generate a set of infant-specific network templates (Table 1). In addition, two independent neonatal datasets with extended acquisition of functional data were used for validation analyses, including rs-fMRI data from Oregon Health and Science University (OHSU, n = 14), and auditory oddball task data from Washington University in St. Louis (WashU, n = 26). All OHSU and WashU infants were born full term (>36 weeks gestational age). All of the pregnancies were uncomplicated with the exception of 5 participants in the OHSU cohort: 4 of the birthing parents experienced vaginal bleeding, and 1 had gestational diabetes. We also used individualized networks derived from 10 subjects available through the Adolescent Brain Cognitive Development Study (ABCD) Community MRI Collection (ABCC) as a comparison to an older age group (Casey et al., 2018; Volkow et al., 2018; Feczko et al., 2021). Analysis of neuroimaging data was approved by the Institutional Review Board at University of Minnesota. For all neonatal cohorts, anatomical and resting-state data were acquired during sleep. A portion of the neonatal subjects were excluded either due to data quality issues or too little resting-state data acquired (see full neonatal dataset demographics in Supplementary Table 1).

**Table 1.** Overview of datasets and demographic information.

	ABCD	UCI**	OHSU	WashU
Subject #	10	69	14	26
Delivery GA (weeks)		39.2 ± 1.5(range 34.6 - 41.9)	40.0 ± 0.7(range 39 - 41.4)	38.2 ± 1.0(range 36 - 39)
Scan age	119 ± 2.4 months	26.2 ± 11.4 days(range 5 - 74)	16.4 ± 6.9 days(range 5 - 30)	27.9 ± 8.9 days(range 11 - 45)
Minutes of fMRI data	Set to 20	5.8 ± 0.5(range 4.2 - 6.4)	38.5 ± 10.3(range 21.5 - 57.1)	31.4 ± 8.3(range 20 - 44.7)
Sex assigned at birth	30% F, 70% M	49% F, 51% M	57% F, 43% M	58% F, 42% M

Values displayed represent the group average ± SD with the range of minimum to maximum in parentheses.

** The UCI dataset was used for generating a set of infant-specific resting-state network templates. The remaining datasets with fewer, highly sampled subjects were used to generate individualized networks.

ABCD, Adolescent Brain Cognitive Development Study; UCI, University of California Irvine; OHSU, Oregon Health & Science University; WashU, Washington University St. Louis; GA, gestational age; fMRI, functional MRI; F, female; M, male (sex assigned at birth).

### 2.2 MRI image acquisition of neonatal datasets

#### 2.2.1 UCI

The dataset acquired at the University of California Irvine includes N = 69 newborn infants (mean age 26.2 days; 34 female, 35 male) with greater than 4.2 minutes of resting-state data post-motion correction (Table 1). Prior to motion correction, the median amount of resting-state data acquired per subject for this sample was 6.5 minutes. fMRI images were acquired on a Siemens 3 T TIM Trio scanner with a 12-channel head coil. For functional imaging, a BOLD gradient-echo, echo-planar sequence was used (32 ascending-interleaved axial 4 mm slices with a 1 mm skip; resolution = 3.4 × 3.4 × 4 mm; TE = 30 ms; TR = 2000 ms; FOV = 220 × 220 × 160 mm; flip angle = 77°). For anatomical reference, a T2-weighted scan (TR = 3200 ms, TE = 255 ms, resolution = 1 × 1 x 1 mm, 4.18 minutes) and a T1-weighted scan (MR-RAGE TR = 2400 ms, inversion time = 1200 ms, TE = 3.16 ms, flip angle = 8°, resolution = 1 × 1 × 1 mm, 6.18 minutes) were acquired. This dataset is further described in Thomas et al. (2019) and Thomason et al. (2015).

#### 2.2.2 OHSU

The dataset acquired at OHSU includes N = 14 newborn infants (mean age 16.4 days; 8 female, 6 male) with greater than 20 minutes of resting-state data after correcting for motion using a frame displacement threshold of 0.3 mm (Table 1). Three out of the 14 subjects were scanned across several days. In order to control for the rapid changes in neural development that occur within the first month after birth, we only included functional data that were collected within the same 9-day period. The average maximal range between scans was 3.6 ± 0.7 days, and a median amount of 57 minutes of data was collected for each subject. fMRI images were acquired at a Siemens Prisma 3 T MRI scanner with a 64-channel head coil. For functional imaging, a BOLD gradient-echo, echo-planar sequence (72 slices, 2.0 mm isotropic resolution, TE = 37 ms, TR = 800 ms, MB factor = 8, FOV = 208 x 208 mm; flip angle = 52°, MB factor = 4) was used. For anatomical reference, a T2-weighted scan (208 sagittal slices, TR = 3200 ms, TE = 564 ms, resolution = 0.8 x 0.8 x 0.8 mm) and a T1-weighted scan (208 sagittal slices, TR = 2400 ms, TE = 2.24 ms, resolution = 0.8 x 0.8 x 0.8 mm, flip angle = 8°, inversion time = 1060 ms) were acquired.

#### 2.2.3 WashU

The dataset, acquired at the Washington University in St. Louis, includes *N* = 26 newborn infants (mean age 27.9 days, 15 female, 11 male) with greater than 20 minutes of resting-state data post-motion correction (Table 1). The subjects were part of the larger cohort described in Sylvester et al. (2021) that were recruited from a separate study at Washington University (Early Life Adversity, Biological Embedding, and Risk for Developmental Precursors of Mental Disorders, i.e., eLABE; https://eedp.wustl.edu/research/elabe-study). fMRI images were acquired on a Siemens 3 T Prisma scanner with a 64-channel head coil. For functional imaging, a BOLD gradient-recalled echo-planar multiband sequence (72 slices, 2.0 mm isotropic resolution, TE = 37 ms, TR = 800 ms, MB factor = 8) was used. A structural T2-weighted scan (208 sagittal slices, TE = 563 ms, TR = 4500 ms, resolution = 0.8 x 0.8 x 0.8 mm) and a T1-weighted scan (TR = 2400 ms, inversion time = 1000 ms, TE = 2.22 ms, flip angle = 8°, resolution = 0.8 × 0.8 x 0.8 mm) were acquired as part of the eLABE study (0–15 days prior, mean =1.1 days). An oddball task paradigm was presented during the BOLD scans with a 400 ms long white noise stimulus, played in irregular intervals (9–14 seconds), marking the deviant sound with respect to the regular scanner background noise. Each BOLD scan lasted for approximately 6 minutes and contained 24 deviant sounds, starting with 56 seconds of regular scanner noise. Depending on the infant’s compliance, up to 11 such scans were collected. A median of 40 minutes of data for each subject were collected. This dataset is further described in Sylvester et al. (2021).

### 2.3 Preprocessing of neonatal datasets

#### 2.3.1 General preprocessing

Neonatal images were preprocessed using the infant-abcd-hcp-pipeline (Sturgeon et al., 2023), a custom modification of the existing HCP pipeline (Feczko et al., 2021; Glasser et al., 2013). For a detailed description of the pipeline, see Supplementary Methods. In brief, the pipeline can be divided into volumetric and surface processing steps. In the volumetric data processing step, anatomical images are segmented and registered to the MNI infant atlas. In the surface processing step, functional data are projected onto the atlas space surfaces. This step additionally includes denoising of functional data and motion censoring using framewise displacement (FD).

#### 2.3.2 Task regression in WashU cohort

In order to generate individualized network maps for the WashU data, we first regressed the auditory oddball task from preprocessed dense time series by using the ABCD-BIDS task fMRI pipeline, abcd-bids-tfmri-pipeline (Feczko et al., 2021; Juliano et al., 2022) to run Level 1 analysis. Responses were modeled using a general linear model (GLM) with separate finite impulse response (FIR) basis functions to model convolution of the BOLD response. Model parameters were chosen following (Sylvester et al., 2021). Separate FIR regressors were used for each of the 40 BOLD frames following white noise onset. The pipeline performed surface-based smoothing by converting the volumetric parameter from Sylvester et al. (2021) (6 mm full width at half maximum Gaussian kernel) to an equivalent sigma value. No temporal smoothing was performed. The first 4 frames of each task run were censored, and motion correction was performed using a framewise displacement threshold of 0.9 mm. The residuals were then used for standard connectivity preprocessing using the infant-abcd-hcp-pipeline starting from fMRIVolume (Sturgeon et al., 2023).

### 2.4 Generation of infant-specific resting-state network templates

Template matching assigns network labels by comparing the voxel-wise whole-brain connectivity of a single subject (Gordon, Laumann, Adeyemo, et al., 2017) to a set of network templates that represent a basic expected spatial topology for each network. For all analyses, the subcortical data were removed from the dense time series in order to perform analyses on cortical data only.

We generated a set of infant network templates, which consists of a separate template for each resting-state network, using 69 UCI subjects with more than 4.2 minutes of resting-state data post-motion correction (FD < 0.3 mm). Seed-based correlation was performed with each of the 14 adult resting-state networks previously generated using Infomap community detection on an average connectivity matrix from 120 subjects (Gordon et al., 2016; Laumann et al., 2015). These networks include: the auditory network (AUD), the default mode network (DMN), the parietal medial network (PMN), the sensorimotor dorsal network (Smd), the ventral attention network (VAN), the cingulo-opercular network (CO), the frontal parietal network (FP), the parietal occipital network (PON), the sensorimotor lateral network (SMl), the visual network (VIS), the dorsal attention network (DAN), the medial temporal network (MTL), the salience network (Sal), and the temporal pole network (Tpole).

The dense time series from each subject is first smoothed using a within-frame spatial Gaussian smoothing kernel of 2.25 mm based on the individual subject’s midthickness surface mesh from Freesurfer. For each subject, an average dense time series, or seed, is created for each network by averaging the dense time series of grayordinates contained with a given network identified from the aforementioned Gordon parcellation (Gordon et al., 2016; Laumann et al., 2015). Each subject-specific network seed is then correlated with the time series of each grayordinate in the brain, resulting in a set of seed-based correlation maps for each set of networks in each subject. The seed-based maps for each network are averaged across subjects and thresholded to 1 SD to preserve only the grayordinates with the highest connectivity. The result is a set of 14 resting-state network templates derived from infant resting-state connectivity using adult resting-state networks as priors for defining the initial seed regions. For comparison, we also generated a cortical version of the adolescent template set from (Hermosillo et al., 2022) using 161 9–10 year old ABCD subjects with at least 10 minutes of low-motion resting-state data (Fig. 2 and Supplementary Fig. 2).

The sets of infant and adolescent network templates were compared visually for notable similarities and differences (Fig. 2). Difference maps were also created (by subtracting the infant from the adolescent network map for each template) to visualize these results in an alternative manner (Supplementary Fig. 3). In addition, we correlated matching non-zero value grayordinates between infant and adolescent templates. In order to visualize the distribution of these correlations, the data points were color-coded by a probability density estimate for each point using Matlab’s *ksdensity* function using normal kernel smoothing (Supplementary Fig. 3).

### 2.5 Generation of individualized resting-state network maps

#### 2.5.1 Template matching

To create individualized network maps utilizing our set of infant network templates for each subject in the testing data (N = 26 WashU, N = 14 OHSU), dense time series were first smoothed using a smoothing kernel of 2.25 mm and motion-corrected with an FD of 0.3 mm, matching the procedure performed with subject data used for template creation as described above. A whole-brain connectivity matrix is then generated by correlating the motion-censored dense time series of each grayordinate against all other grayordinates. To assign each grayordinate to a network, we calculated an eta^2^ value to determine the similarity of the connectivity pattern of each grayordinate to the connectivity pattern of each of the 14 networks in our set of infant network templates. Each grayordinate is then assigned to the network with the highest eta^2^ value, resulting in an individualized resting-state network map for each subject in our testing datasets.

#### 2.5.2 Infomap

For comparison with the template matching approach, we also generated individual network maps using Infomap, a strictly data-driven analysis, in the OHSU cohort following the same procedures as Gordon, Laumann, Gilmore, et al. (2017) and Power et al. (2011). In contrast to template matching, which uses a fixed set of network templates to apply a supervised set of assignments, Infomap is an unsupervised community detection algorithm which identifies any number of networks using information flow (Rosvall & Bergstrom, 2007, 2008). The same whole-brain connectivity matrices generated for template matching were also used for Infomap. To control for effects of spatial smoothing, we set a minimum distance of 20 mm, meaning that grayordinates located within 20 mm were excluded from the list of connections. We then generated a set of thresholded connectivity matrices using a range of tie densities (0.3%, 0.4%, 0.5%, 1.0%, 1.5%, 2.0%, 2.5%, and 3.0%) so that only connections that have higher than the given tie density/percentage connectivity are assigned to networks. Infomap identifies network assignments for each of these connectivity matrices based on an optimized Hamming code length using a flow-based method (Rosvall & Bergstrom, 2007, 2008). Any networks with fewer than 400 grayordinates were relabeled as “unassigned.” In order to create a “consensus” network map across the network maps generated at various tie densities, all grayordinates are first assigned based on the most stringent threshold (0.3%). The unassigned grayordinates are then assigned based on the network maps generated with the 0.4% tie density, and so forth, until all grayordinates are assigned to a network. Additionally, we set a minimum region size of 30 grayordinates so that contiguous network clusters smaller than this are removed and reassigned to the largest neighboring network. Finally, to facilitate split-halves analysis and comparison with template matching network maps, we employed a Jaccard similarity threshold of 0.2. This threshold was applied to reassign the arbitrary Infomap labels to the networks that were most similar to the 14 adult resting-state network parcellations generated using Infomap community detection on a large adult sample mentioned earlier (Gordon et al., 2016; Laumann et al., 2015).

### 2.6 Split-halves reliability analysis

**Fig. 1.**
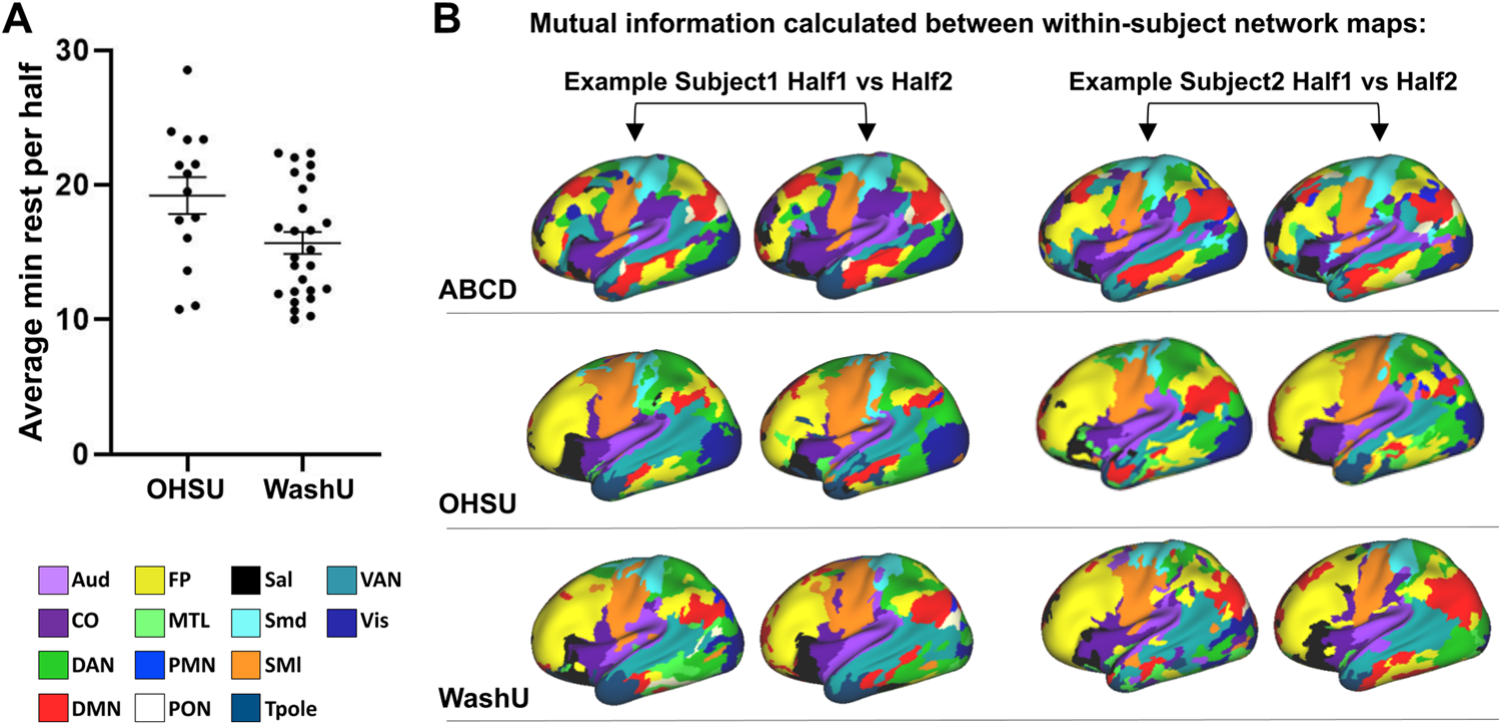
Split-halves reliability analysis. (A) Average minutes of resting-state data in split halves in infant subjects (note that the number of minutes contained in each half is not necessarily equal due to variation in the number of frames included after motion correction). (B) Two examples per cohort of individual network maps generated from the first and second halves of split resting-state time series using template matching. Normalized mutual information is used to compare the similarity of the networks generated from the first versus second half of each subject’s functional data and compared to a null distribution generated from comparing inter-subject network maps. Aud, auditory network; DMN, default mode network; PMN, parietal medial network; Smd, sensorimotor dorsal network; VAN, ventral attention network; CO, cingulo-opercular network; FP, frontal parietal network; PON, parietal occipital network; SMl, sensorimotor lateral network; VIS, the visual network; DAN, dorsal attention network; MTL, medial temporal network; Sal, the salience network; Tpole, temporal pole network.

In order to validate whether template matching can reliably generate resting-state networks specific to individual infants, we performed split-halves reliability analysis on the OHSU and WashU neonatal datasets where subjects have at least 20 minutes of low-motion resting-state data (FD = 0.3) (Fig. 1A). In this analysis, separate network maps are generated from the first and second half of each subject’s time series data and the similarity of the networks is compared using normalized mutual information (NMI) (Fig. 1B). We also performed this analysis on Infomap-generated networks in the OHSU cohort.

### 2.7 Statistical analysis and reporting

All NMI groups were tested for normality (using Kolmogorov-Smirnov) and equal variance (F-test used when comparing two groups and Brown-Forsythe and Bartlett’s tests used when comparing more than two groups) before using parametric tests. Parametric tests included t-tests for comparing two groups and one-way ANOVA followed by Tukey’s test for post hoc comparisons to compare more than two groups. In the event that data were normally distributed but did not satisfy the assumption of equal variance, a t-test with Welch’s correction was used for comparing two groups and Welch ANOVA tests followed by post hoc t-tests with Welch’s correction for comparing more than two groups. If data were not normally distributed, Mann Whitney and Kruskall-Wallis (followed by Dunn’s post hoc comparison) tests were applied to two-group and greater than two-group comparisons respectively. The specific statistical test used for each comparison is noted in the Results section. Unless standard deviation is specified, averages are reported in both the text and figures with standard error.

## 3 Results

### 3.1 Infant network templates include all large-scale adult brain networks

**Fig. 2.**
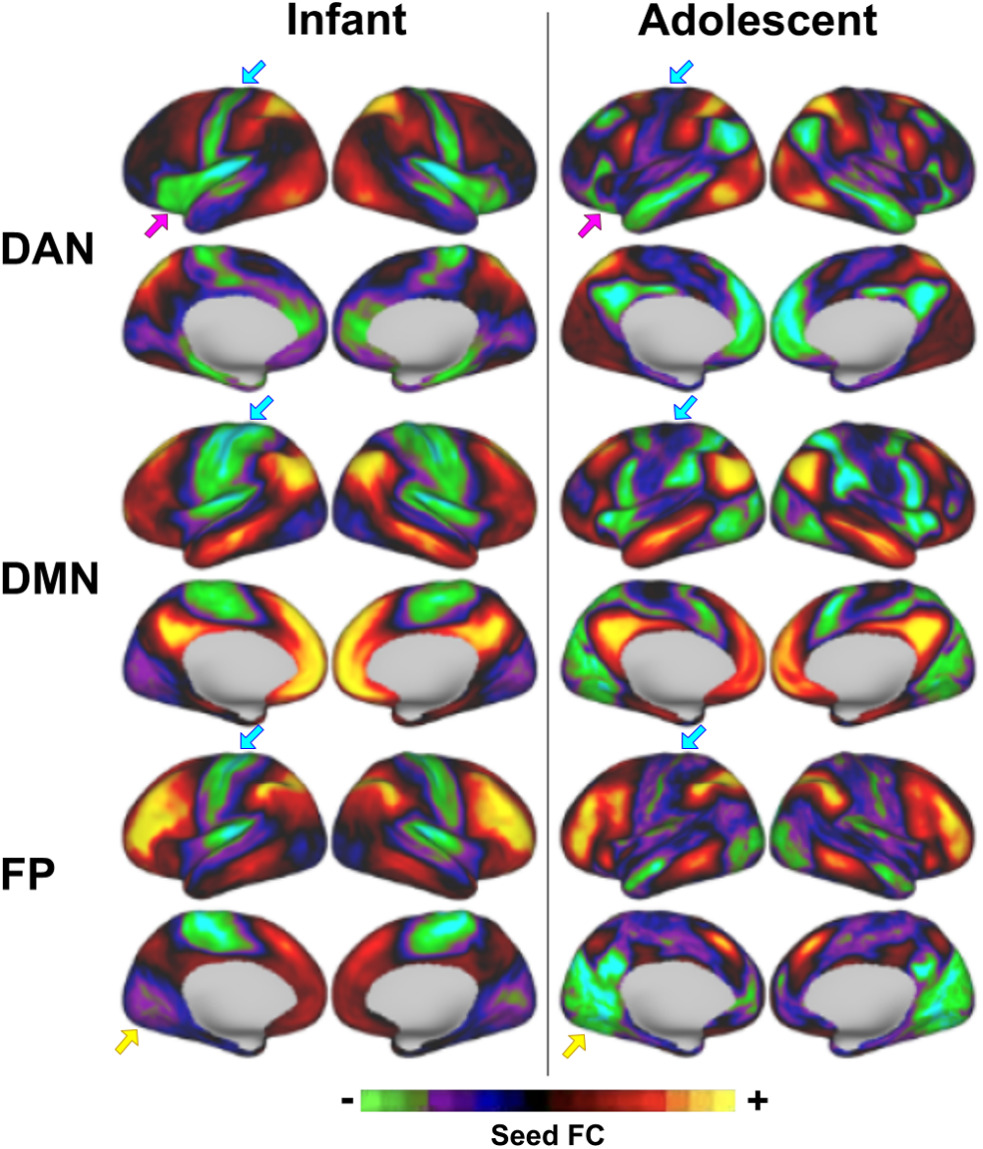
Comparison of select infant versus adolescent network templates. All major adult networks were observed in the infant template. The color bar displays the strength and valence of functional connectivity (FC) between a given seed region and the rest of the brain. Though the templates are initialized with the same network maps, the infant template differs from the adolescent template in several ways. One striking difference was the strong anticorrelation with the sensorimotor region present in infants, particularly evident in DAN, DMN, and FP (blue arrows). Other notable differences with these particular infant templates are the relatively strong anticorrelation between DAN and insula (fuchsia arrows) and between FP and the visual cortex (yellow arrows).

The set of infant network templates generated from the neonatal UCI cohort (Supplementary Fig. 1) comprised all major networks identified in adults (Supplementary Fig. 2). Direct comparison to the adolescent network templates revealed clear differences in patterns of connectivity (Fig. 2), the most striking of which was a strong anticorrelation between the sensorimotor region and multiple networks including DMN, DAN, and FP (see Supplementary Fig. 3 for alternative visualization and further statistical comparisons). For these network templates, we also visualized the connectivity of relevant brain regions within the average UCI dense connectivity matrix to illustrate that the infant network templates do not contain artificial connections as a result of using the adult resting-state networks as priors (Supplementary Fig. 4).

### 3.2 All major adult networks are reliably identifiable in individualized neonatal resting-state network maps with limited amounts of resting-state data

Using the set of infant resting-state network templates (Supplementary Fig. 1), we performed template matching to generate individualized resting-state network maps for each subject from three independent neonatal datasets, including UCI, OHSU, and WashU (Supplementary Fig. 5). We also generated network maps using the adolescent network templates and found that the resulting network maps were highly similar regardless of which template was used (Supplementary Fig. 6). This suggests that the network assignments were driven largely by the individual subject data as opposed to the age of the template subjects. It could also indicate that the set of infant resting-state network templates are largely similar to the adolescent templates. We therefore used the set of infant network templates as a basis for all subsequent analyses.

**Fig. 3.**
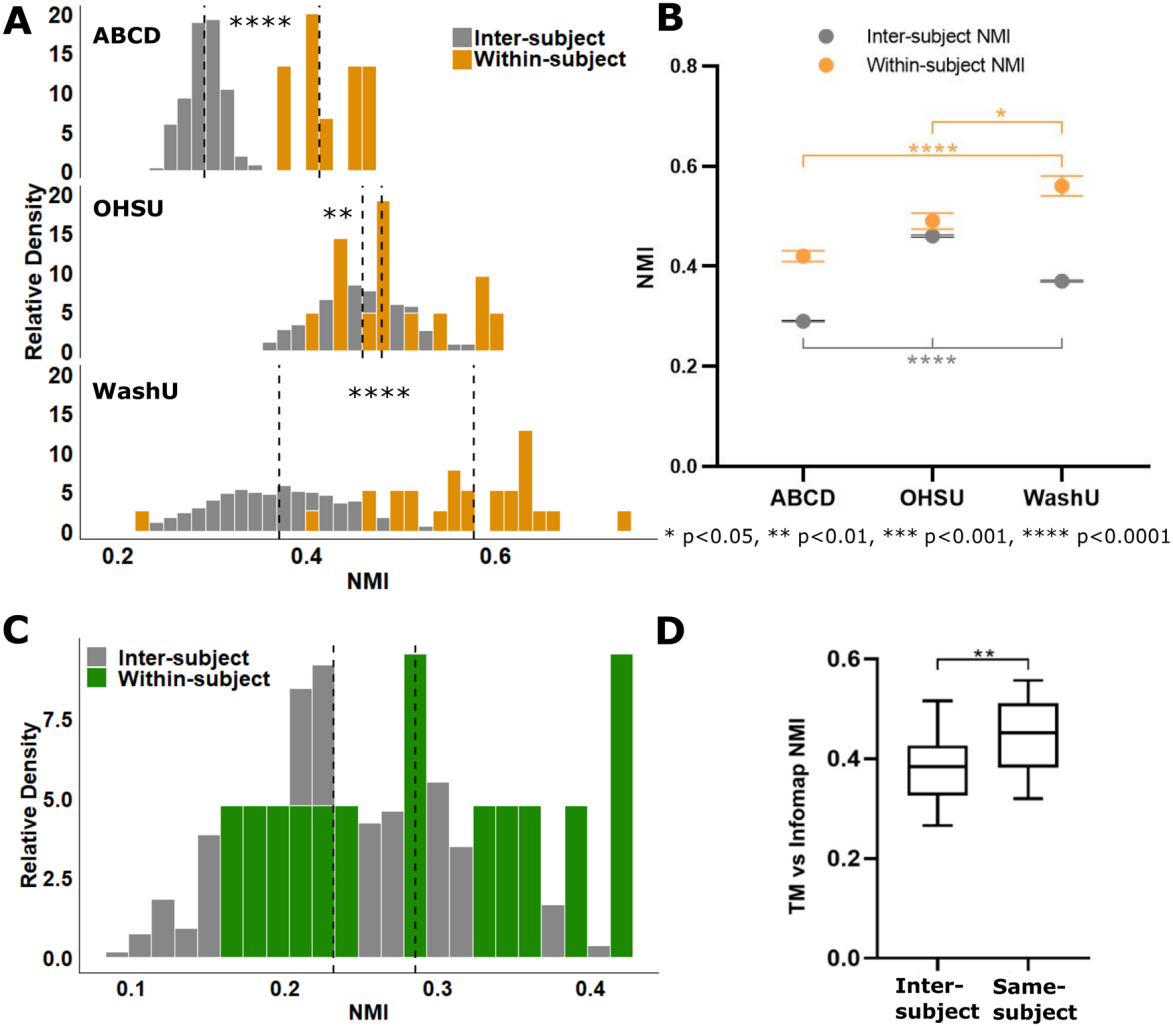
Normalized mutual information (NMI) used to quantify the similarity of network maps derived from split-halves reliability analysis and across methods (template matching vs. Infomap). (A) Using template matching, the similarity of within-subject network maps was significantly greater than inter-subject similarity for both adolescent and infant cohorts, indicating that template matching effectively resolves individualized network maps in infants. (B) Population statistics comparing the similarity of within- and inter-subject network maps across cohorts. Interestingly, infant network maps were generally more similar across individuals (i.e., inter-subject NMI was higher) compared to adolescents. (C) Contrary to template matching, Infomap-derived network maps did not show a significant difference between within- and inter-subject network similarity with split-halves analysis. (D) Direct comparison of network maps from TM versus Infomap derived from the full amount of resting-state data available for each infant (OHSU cohort only): same subject network maps compared between methods were significantly more similar than inter-subject network maps. *p < 0.05, **p < 0.01, ***p < 0.001, and ****p < 0.0001.

Split-halves reliability analysis (Fig. 1) was performed to compare the similarity of within- versus inter-subject individualized network maps generated from each cohort (Fig. 3A). One would expect that if the template matching technique is robust (i.e., the resultant networks are indeed individual-specific), then the network maps generated from two halves of the same subject should be more similar than the network maps generated from different subjects. As previously reported for adolescents, the similarity of within-subject network maps (0.42 ± 0.01 NMI) was significantly greater than the similarity of inter-subject network maps (0.29 ± 0.00 NMI) (t = 11.87, df = 9.313, p =< 0.0001, t-test with Welch’s correction). The neonatal cohorts displayed the same outcome: within-subject network similarity for both OHSU (0.49 ± 0.02 NMI) and WashU (0.56 ± 0.02 NMI) was significantly greater than inter-subject network similarity (0.46 ± 0.002 and 0.37 ± 0.002 NMI respectively) (OHSU: t = 2.884, df = 376, p = 0.0041, t-test; WashU: U = 2156, p < 0.0001, Mann-Whitney U). In the WashU cohort, individualized network maps could also be generated from the task data prior to regression of auditory oddball responses despite the additional signal and noise introduced by the task (Supplementary Fig. 7A). However, the within-subject network similarity was significantly lower by an average of 0.16 NMI compared to results derived from task-regressed data (W = 349.0, p < 0.0001, Wilcoxon matched-pairs signed rank test; Supplementary Fig. 7B), indicating that task regression effectively aided in the delineation of individualized networks. We also assessed the impact of spatial smoothing on split-halves reliability analysis using the OHSU cohort and confirmed that individual differences could still be resolved despite the increased noise in the resulting network maps (within-subject NMI 0.36 ± 0.02; inter-subject NMI 0.31 ± 0.003; t = 2.46, df = 376, p = 0.0143, t-test). In sum, our results indicate that template matching can effectively be applied to infants to generate individual-specific networks.

We also observed differences in average similarity values between cohorts (Fig. 3B). Infants showed significantly greater inter-subject similarity compared to adolescents (H(2) = 672.1, p < 0.0001, Kruskal-Wallis; p < 0.0001 for each post hoc comparison). Between infant cohorts, the OHSU infants, which were scanned at a significantly younger age (16.4 ± 6.9 days) compared to WashU infants (27.9 ± 8.9 days) (t = 4.2, df = 38, p = 0.0002, t-test; Table 1), had significantly greater inter-subject similarity compared to WashU in addition to adolescents (p < 0.0001 post hoc comparison). Though these group differences could be caused by a variety of factors, it is interesting that younger ages generally resulted in greater network similarity between subjects. However, it should be noted that, though OHSU infants were scanned at an earlier age, their gestational age was greater than WashU (U = 6, p < 0.0001, Mann-Whitney U; Table 1) and there was no significant difference between the sum of scan and gestational age, that is, postmenstrual age. Within-subject similarity also showed differences across groups (F(2,47) = 2.5, p = 0.0001, one-way ANOVA): the WashU cohort’s within-subject network similarity was higher than that for both ABCD (p < 0.0001) and OHSU (p = 0.045).

We also performed split-halves analysis with individualized network maps derived from Infomap, the current gold standard for community detection of resting-state functional networks (Gates et al., 2016; Lancichinetti & Fortunato, 2009). Though there was a slight positive shift in the similarity of within- (0.29 ± 0.02 NMI) versus inter-subject (0.24 ± 0.003 NMI) similarity, the difference was not significant, indicating that more data are required to generate individualized network maps than the amount used for template matching (Fig. 3C; U = 1786, p = 0.0577, Mann-Whitney U). Comparing the Infomap- to template matching-derived network maps directly, the similarity of same-subject network maps (0.44 ± 0.02 NMI) was significantly higher than inter-subject (0.38 ± 0.01 NMI) (Fig. 3D; U = 668, p = 0.0024, Mann-Whitney U). This suggests that template matching produces similar individualized network maps to Infomap’s purely data-driven algorithm, but is able to resolve individual differences with a lower amount of resting-state data.

Though template matching reliably defined individual resting-state network maps in infants, there was more variability and overlap in the distributions of within- and inter-network similarity compared to adolescents (Fig. 3A). It is possible that individual specificity is simply more difficult to assess in infants if network maps are generally less differentiated between subjects at earlier stages of development. However, it is also likely that infants simply require larger amounts of resting-state data to generate highly topologically precise individualized networks due to inherently lower signal-to-noise ratio (SNR) of infant data compared to adults. We therefore asked whether the template matching-derived network maps showed some level of topological precision in addition to being individualized.

### 3.3 Infants likely require upwards of 25 minutes of resting-state data to generate high-precision network maps

The split-halves analysis demonstrated, in two independent neonatal cohorts, that template matching can effectively identify individual-specific networks in infants. However, the topological precision of the resultant networks and how much resting-state data are required to achieve network maps that are not only individualized, but also highly precise, are still open questions.

**Fig. 4.**
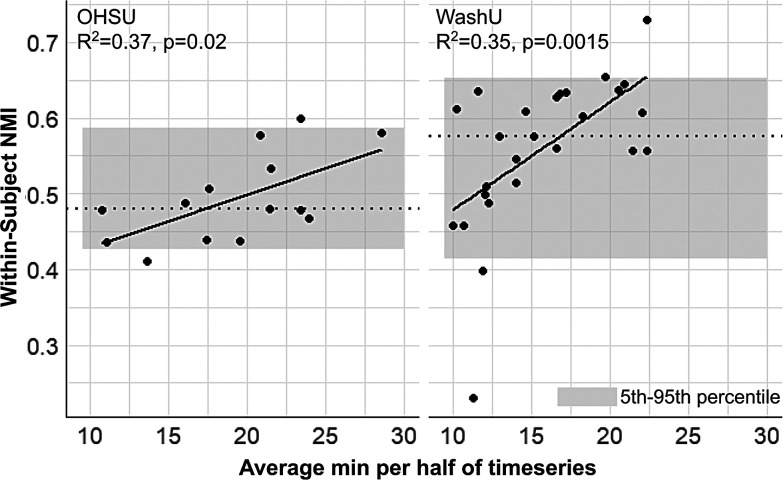
Within-subject network map similarity is significantly associated with the number of minutes of rest available per subject. The plots show a significant positive linear correlation between the average minutes of rest per half for each subject and within-subject NMI in OHSU (left) and WashU (right) cohorts. Gray boxes are 5th to 95th percentiles of the distribution of within-subject network map similarity; dashed lines are group-average NMIs.

To analyze the split-halves reliability results further, we first performed a simple linear regression between the average amount of resting-state data available between halves for each subject and the associated within-subject network map similarity (NMI). There was a significant positive relationship in both OHSU (R^2^ = 0.37, p = 0.02) and WashU (R^2^ = 0.35, p = 0.0015) cohorts, demonstrating that within-subject reliability increases as the amount of data available increases (Fig. 4). This also indicates that the higher variability in within-subject network map similarity in infants (Fig. 3A) may be due in part to the variable amount of resting-state data available across individuals in these cohorts (Table 1).

**Fig. 5.**
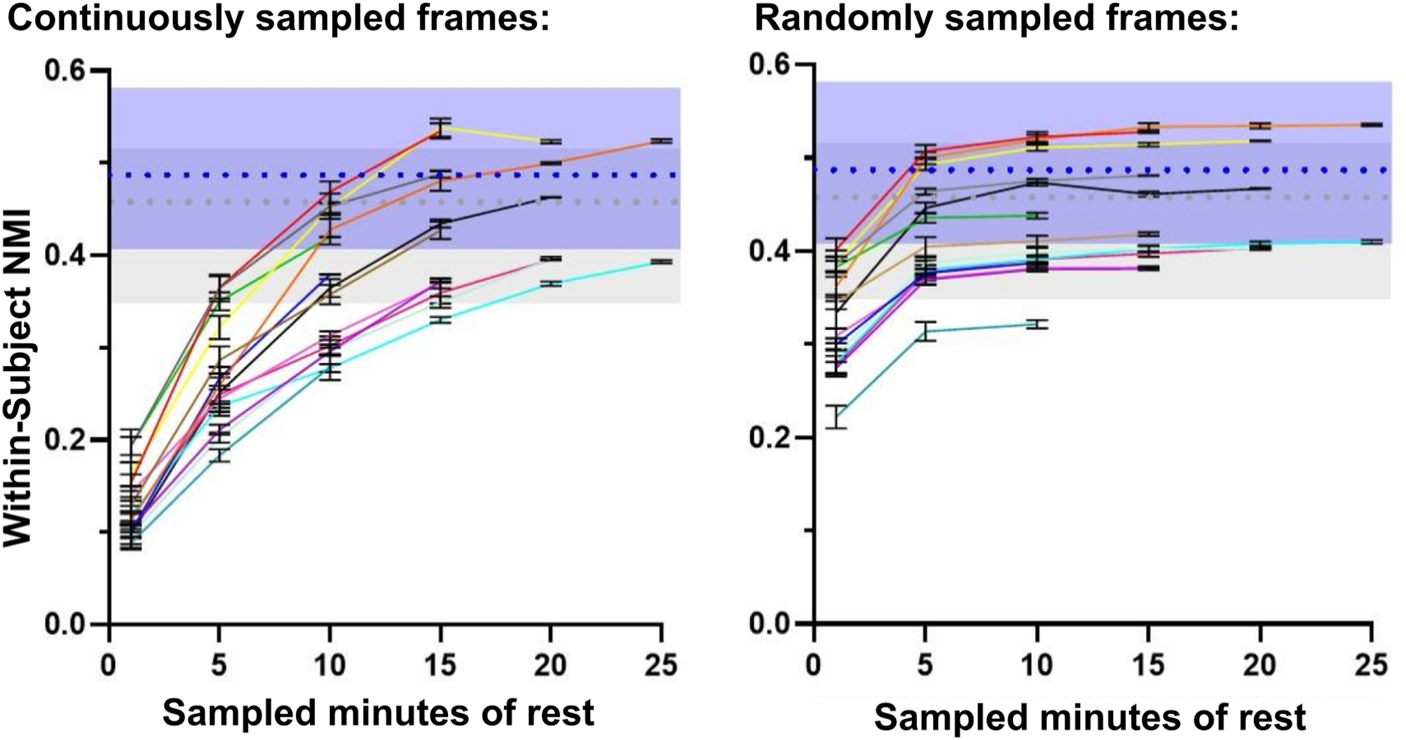
Similarity of within-subject network maps generated from various intervals of resting-state data. For each subject, network maps were generated from continuously (left plot) and randomly (right plot) sampled data in 1-, 5-, 10-, 15-, and 25-minute segments from the first half of the dense time series (averaged over 10 repetitions). The resulting network maps were compared to the network maps derived from the full second half of the time series. The blue boxes display the 5th to 95th percentiles (0.43 and 0.59 NMI) of group-wide within-subject similarity values; the gray boxes display the same for group-wide inter-subject similarity (0.38 and 0.53 NMI); and the average NMIs are displayed as dashed lines. Each color-coded line in the plots represents data from an individual subject in the sample.

To control for the variable amount of resting-state data available between infants and assess how much data are required to generate high-precision network maps, we performed a modified version of the split-halves analysis in the OHSU cohort in which the first half of the resting-state data was sampled at various intervals (using either randomly- or continuously sampled frames) to compare to networks derived from the holdout half (Fig. 5). As Hermosillo et al. (2022) displayed with adolescent data, one would expect to see the maximal within-subject NMI plateau at the amount of resting-state data that is required to generate high-precision maps, beyond which addition of more data does not significantly impact within-subject similarity. In the infant cohort, though individual within-subject similarity does appear to plateau for a portion of subjects when using randomly sampled frames, they do not plateau using continuously sampled frames. Note that higher within-subject similarity with random frame sampling is expected due to the reduction of temporal autocorrelation (Hermosillo et al., 2022; Laumann et al., 2015). Furthermore, the results with random frame sampling are inconsistent between individuals and multiple subjects plateau either below or within the 5th percentile of group-wide inter-subject NMI, which is the lower range of network similarity expected purely by chance. These results suggest that infants likely require upwards of 25 minutes of resting-state data to generate network maps that are highly precise. A greater amount of data and/or data with higher signal-to-noise ratio is therefore needed to address how much data are needed for high-precision network mapping in infants.

## 4 Discussion

This study suggests that all major large-scale resting-state networks identified in adults (Hermosillo et al., 2022) can be reliably detected in individual infants via application of template matching. Furthermore, by comparing the similarity of networks generated from the same versus different subjects, we found that the topology of network maps was individual-specific. Our findings also indicate that, though the network maps are individual-specific, there is potential for increasing precision. Specifically, there was no plateau in the similarity of within-subject network maps with increasing amounts of resting-state data up to 25 minutes. There is a clear need to push boundaries in data collection techniques that enhance signal-to-noise ratio such as multi-echo acquisition (Lynch et al., 2020) as well as longer acquisition times. These advancements will empower the field to harness the full potential of precision mapping in infants to establish a clinically robust understanding of how early functional connectivity relates to subsequent behavioral phenotypes and health outcomes (Gratton et al., 2022; Kraus et al., 2023).

### 4.1 Reliable identification of adult resting-state networks in infants

Our set of infant network templates correspond to canonical resting-state networks identified in adults (Fair et al., 2008; Gordon et al., 2016; Miranda-Dominguez et al., 2018; Power et al., 2011; Smith et al., 2015; Yeo et al., 2011). Though these large-scale networks may have similar spatial topography to adults, there are clear differences in certain features of connectivity. For instance, in contrast to the early adolescent network templates generated from ABCD (Supplementary Fig. 2), several infant networks (including DMN, DAN, and FP) show distinct negative correlations with the sensorimotor region (Fig. 2). This is congruent with prior work demonstrating that the topography of positive functional connectivity is much more similar between neonates and adults than negative connectivity (Sylvester et al., 2022).

Unique features of functional connectivity in neonates may reflect the staged development of functional networks (Cadwell et al., 2019; Doria et al., 2010; Eyre et al., 2021; Moore et al., 2023). For example, the developing motor system displays highly irregular patterns of temporal neuronal activity thought to underlie behavioral variability necessary for learning and complex behaviors (Darshan et al., 2017; Hadders-Algra, 2010). The anticorrelation with sensorimotor regions observed in several of the infant network templates could reflect active suppression of this activity to enable refinement of established connections and formation of topographically precise motor maps. The differences in infant connectivity could also be attributed to data acquisition during natural sleep compared to the awake state (Graham et al., 2015). However, canonical resting-state networks are preserved and show largely similar patterns across wake and sleep states in adults, and, as such, it is unlikely that the findings outlined here are solely due to sleep state (Boly et al., 2008; Horovitz et al., 2009; Larson-Prior et al., 2009; Picchioni et al., 2013; Tagliazucchi et al., 2013). Ongoing research on fMRI conducted in infants in sleep and wake states and comparison to adults will be critical to parse these distinctions further (Mitra et al., 2017; Yates et al., 2023). Future research will need to interrogate further the similarities and differences of neonatal versus adult network connectivity and function to track changes from birth to adolescence. The beginning of the newly started HEALthy Brain and Child Development (HBCD) Study will likely assist in this trend (Volkow et al., 2021).

Using our set of infant network templates as priors for template matching, we reliably detected all major adult networks in individualized network maps of subjects from three independent neonatal datasets. Prior studies have shown considerable variation in the number, topography, and connectivity of networks detected in infants. Networks for somatomotor, primary auditory, primary visual, and extrastriate visual cortex have been identified fairly consistently in early infancy (Fransson et al., 2007; Gao, Alcauter, Smith, et al., 2015; W.-C. Liu et al., 2008; Smyser et al., 2010). Further work suggests that adult dorsal attention (Gao et al., 2013), salience (Alcauter et al., 2015; Gao, Alcauter, Elton, et al., 2015; Gao, Alcauter, Smith, et al., 2015), and frontoparietal networks exist in infants as well (Gao, Alcauter, Elton, et al., 2015; Gao, Alcauter, Smith, et al., 2015), but only in primordial forms. However, more recent evidence demonstrates that robust connectivity gradient organization for the majority of canonical resting-state networks can already be detected *in utero* at 25 weeks gestation, suggesting that the essential architecture of resting-state networks is already formed before birth (Moore et al., 2023). As such, more nascent networks may simply be more difficult to detect. Indeed, recent evidence shows that tuning Infomap to detect long-range connectivity identifies communities with adult-like patterns of connectivity that are missed when using the standard Infomap algorithm (Sylvester et al., 2022). Our results highlight template matching as a potentially robust method for large-scale network detection across development, including during the early critical period of neonatal brain development.

### 4.2 Potential application for personalized precision mapping in infants

Employing personalized precision mapping, built upon extensively sampled individual subjects and study designs tailored to maximize signal and minimize noise, promises to unlock new avenues of research. This approach facilitates an in-depth exploration of both anatomical and functional brain architecture (Braga et al., 2019; Braga & Buckner, 2017; Newbold et al., 2020; Reznik et al., 2023; Sylvester et al., 2020; Xue et al., 2021), offering substantial potential to advance the field in assessing phenotypic diversity and clinical relevance (Gratton et al., 2022; Kraus et al., 2023). The results of the current study build on recent research demonstrating that resting-state networks are already highly individualized in infants (Molloy & Saygin, 2022). However, prior research is variable regarding both the robust detection of resting-state networks as well as the similarity of these networks to those in adults. Sylvester et al. (2022) found that brain maturity, measured by comparing the similarity of the average infant connectivity matrix to average adult data, is actually dependent on data quantity. This suggests that prior findings could be confounded by limited amounts and/or quality of data. Applying personalized precision mapping to infants offers a means to address the inherent noise in infant data compared to adults without relying on group averaging (Smyser & Neil, 2015). This approach empowers the investigation of individual differences throughout development, potentially granting researchers unparalleled insight into the isolated influences of genetics and prenatal factors on network function, while minimizing the impact of childhood environmental factors and life experiences.

Template matching is well suited for precision mapping as well as to accommodate unique characteristics of infant functional connectivity. As discussed in the Introduction, compared to approaches that involve clustering algorithms, it offers a more robust approach for identifying long-range connectivity that has classically been difficult to detect in infants compared to short-range connectivity. In addition, infants display a unique pattern of negative functional connectivity that is very distinct from adults (Supplementary Fig. 3) (Sylvester et al., 2022). Methods that only take positive functional connections into account, including non-negative matrix factorization (Cui et al., 2020; Li et al., 2017) and graph approaches like Louvain (Blondel et al., 2008), may therefore miss important features of infant resting-state functional networks. Template matching is also useful because it relies on a set of network templates that can be applied to different datasets. By contrast, some methods require that both the group-level parcellation schemas and individualized networks come from the same dataset (Shen et al., 2013; D. Wang et al., 2015). These group network solutions are not generalizable to other datasets and are also likely overfit to the test sample, making it harder to capture small variations further from central tendencies, which would be a missed opportunity to fully leverage the power of precision mapping. Furthermore, defining group-level networks from the same data set requires a large number of subjects to be set aside for group-level network solutions, which may limit applicability to precision medicine techniques involving highly neuroanatomically targeted interventions for which the sample size is N = 1.

#### 4.2.1 Individual specificity of network maps

We examined individual specificity of infant networks in two independent neonatal cohorts with at least 20 minutes of low-motion resting-state data. As with adolescents, template matching-derived network maps showed significant within-subject network similarity in both cohorts. We further confirmed that Infomap was not able to generate individualized network maps with as little data as template matching. Our findings suggest that template matching is a robust technique to reliably detect all major adult networks in individualized infant network maps generated from relatively modest amounts of rs-fMRI data.

#### 4.2.2 Precision of network maps

The OHSU and WashU datasets with highly sampled individual neonates provided a unique opportunity to explore high-precision network mapping with maximal topological stability in infants. Prior work in both adults (Gordon, Laumann, Gilmore, et al., 2017; Greene et al., 2020) and adolescents (Hermosillo et al., 2022) shows that within-subject network similarity plateaus with sufficient minutes of resting-state data: at some point, more data does not provide enough additional information to appreciably increase the precision of individually generated network maps. Hermosillo et al. (2022) showed that, using network maps generated with template matching in adolescents, within-subject network map similarity substantially exceeds inter-subject network similarity with as little as 2 minutes of data and plateaus by 10 minutes. In contrast, we did not observe a similar plateau in infants (Fig. 5), indicating that infants may require upwards of 25 minutes of resting-state data and/or data with a higher signal-to-noise ratio to generate individual networks with maximal topological precision.

### 4.3 Challenges and future directions

Infant fMRI data are inherently noisier than adult data, and precision mapping with a focus on maximizing signal-to-noise will be critical to fully resolve the development of infant resting-state networks. The source(s) of reduced signal-to-noise in infants requires further exploration. Acquisition during natural sleep, which is known to shift BOLD oscillations from modular to global in adult sleep, is one highly likely candidate given the similarities observed between functional connectivity in infant and adult sleep (Horovitz et al., 2009; Spoormaker et al., 2012; Yates et al., 2023). Other possible sources include increased spontaneous neural firing, reduced hemodynamic coupling (Hendrikx et al., 2019), increased partial volume averaging effects, or some combination of these variables. One interesting possibility is that precision itself is more difficult to interrogate in infants because individual brains are less differentiated and perhaps more similar compared to later stages of development (Hu et al., 2022). This lack of differentiation could possibly explain why our analysis showed that the similarity of network maps between infant subjects was significantly higher than between adolescents (Fig. 3B).

In order to disentangle these possibilities, approaches to maximize signal-to-noise in infant data will be critical. There is a need and opportunity for both longer data acquisition as well as acquisition protocols that increase signal quality and reliability. Multi-echo acquisition requires significantly less data compared to single-echo to achieve reliable functional connectivity-based measurements within individuals (Lynch et al., 2020; Posse et al., 1999). Additionally, the implementation of NORDIC denoising to decrease thermal noise has emerged as an effective strategy to enhance signal quality (Dowdle et al., 2023; Vizioli et al., 2021). Advances in ultra-high field neuroimaging is another promising path, though a balance must be struck between achieving high spatial resolution and maintaining the essential temporal resolution required for measure whole-brain connectivity (De Martino et al., 2011; Raimondo et al., 2021). These approaches still need to be evaluated in infants, but optimizing signal-to-noise at the stage of acquisition may be critical to addressing the unique challenges of precision functional mapping at early ages.

## 5 Conclusions

This study demonstrates a robust methodological approach for reliably identifying individualized resting-state networks in infants that capture the canonical set of resting-state networks defined in adults. Prior work has demonstrated that variability in the strength of functional network connection in infancy not only relates to both environmental and genetic influences, but is also relevant for subsequent development and risk for neurodevelopmental disorders (Emerson et al., 2017; Graham et al., 2015; Hazlett et al., 2012, 2017; Langer et al., 2017; Rudolph et al., 2018). However, individual topological differences are often averaged out to make group comparisons. Template matching enables us to (1) consistently detect all major functional brain networks already present in the neonatal period and (2) reliably generate individualized network maps in infants. Though we show that the individualized network maps generated with template matching are specific to each subject, the extent of the individual differences remains unclear as we see continued improvement in the similarity of within-subject network maps with greater amounts of resting-state data without a plateau. This study thus provides a critical foundation for future research to identify individualized network maps in infants that are not only reliable, but also highly topologically precise. Such work represents a novel direction in infant functional neuroimaging, with implications for improving understanding of individual differences in neurodevelopment.

## Supplementary Material

Supplementary Material

## Data Availability

This study was conducted with study data provided directly by the study authors. The adolescent data used in this study are part of the Adolescent Brain and Cognitive Development Study: imaging data from this cohort are available via the National Institute of Mental Health Data Archive. The neonatal data used were provided directly by the study authors and are not available for release, but may possibly be made available upon request given the consent of the original study authors. The set of age-specific resting-state network templates generated for the adolescent and neonatal data sets as well the individualized network maps generated across datasets are available upon request. All of the code used for generating the set of resting-state network templates, performing template matching to generate individualized network maps, and comparing network map similarity using normalized mutual information is available for public use: https://github.com/DCAN-Labs/compare_matrices_to_assign_networks. The MRI processing pipeline (used to process the neonatal datasets) and task pipeline are publicly available for download: https://github.com/DCAN-Labs/infant-abcd-bids-pipeline and https://github.com/DCAN-Labs/abcd-bids-tfmri-pipeline
