# Supplementary material for: Towards personalized precision functional mapping in infancy
Source: Imaging Neurosci (Camb). 2024 May 10;2:imag-2-00165. doi: 10.1162/imag_a_00165 (PMC11899874; doi:10.1162/imag_a_00165)
Supplement: Supplementary Material [file imag_a_00165-supp.pdf]

Supplementary Material

Supplementary Results

Supplementary Table 1. Demographic information for full neonatal datasets (population statistics displayed as average ± SD)

	UCI	OHSU	WashU
**Total Subject #**	102	19	43
**# Excluded Subjects**	33	5	17
**Delivery GA (weeks)**	39.2 ± 1.4<br>(range 34.6 - 41.9)	39.9 ± 0.8<br>(range 37.9 - 41.4)	38.2 ± 1.0<br>(range 36 - 40)
**Scan age (days)**	25.4 ± 12.1<br>(range 5 - 64)	15.2 ± 6.5<br>(range 5 - 30)	28.4 ± 9.6<br>(range 11 - 55)
**Sex assigned at birth**	47% F, 53% M	63% F, 37% M	60% F, 40% M
**Reason for subject exclusion**	1	2	1,2

(1) Failed pre- or post-processing quality assessment
(2) Less than 20 minutes of resting state data obtain post-motion correction (FD>0.3)

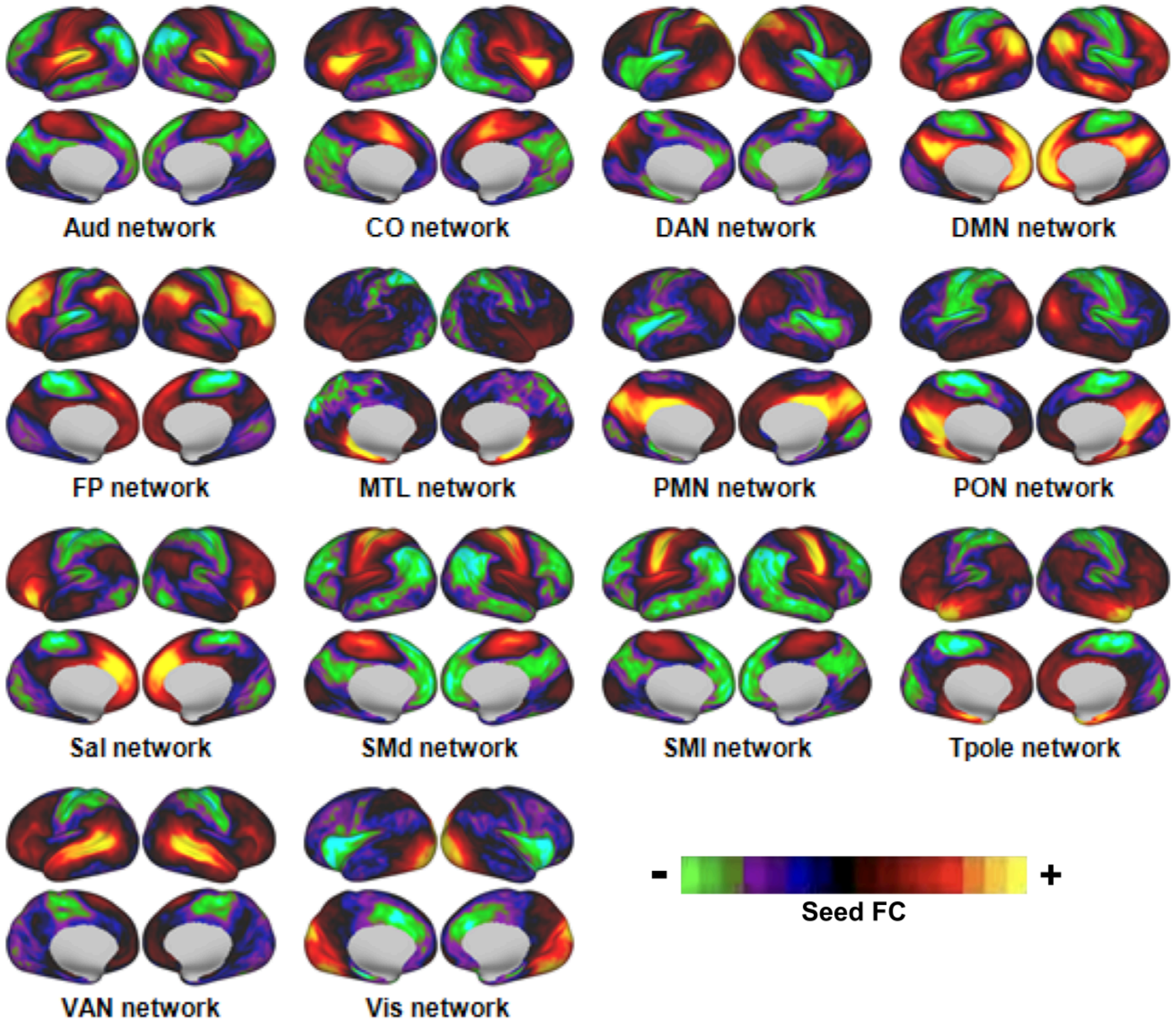

Supplementary Figure 1. Set of infant network templates generated from cerebral cortical dense time series data. Infant templates were generated from 69 UCI neonates with greater than 4.2 minutes of resting state data post-motion correction (FD=0.3).

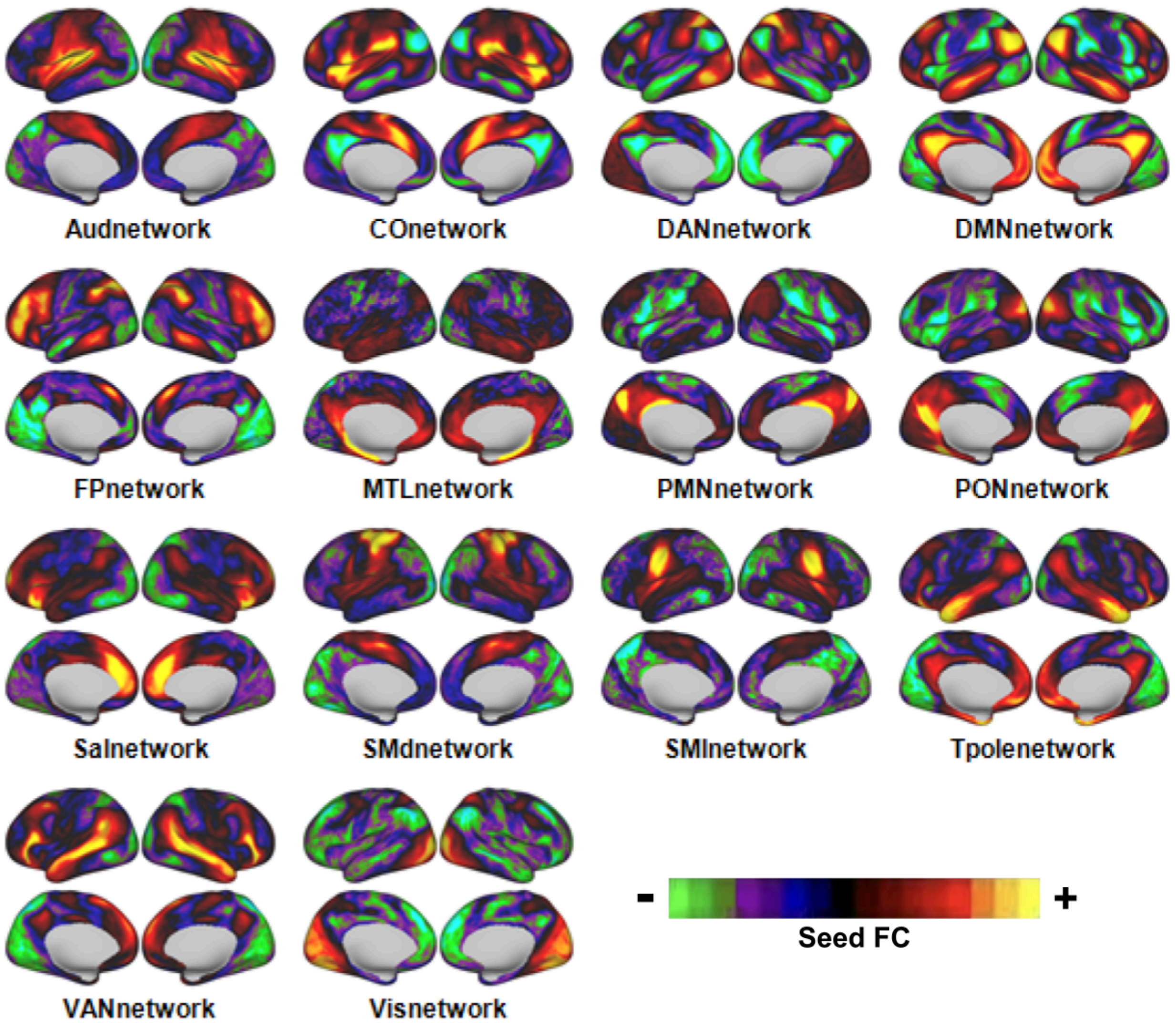

Supplementary Figure 2. Set of early adolescent network templates generated from cerebral cortical dense time series data. This set of early adolescent templates was generated from 161 ABCD subjects (9-10 years old) with greater than 10 minutes of resting state data post-motion correction (FD=0.3)**.**

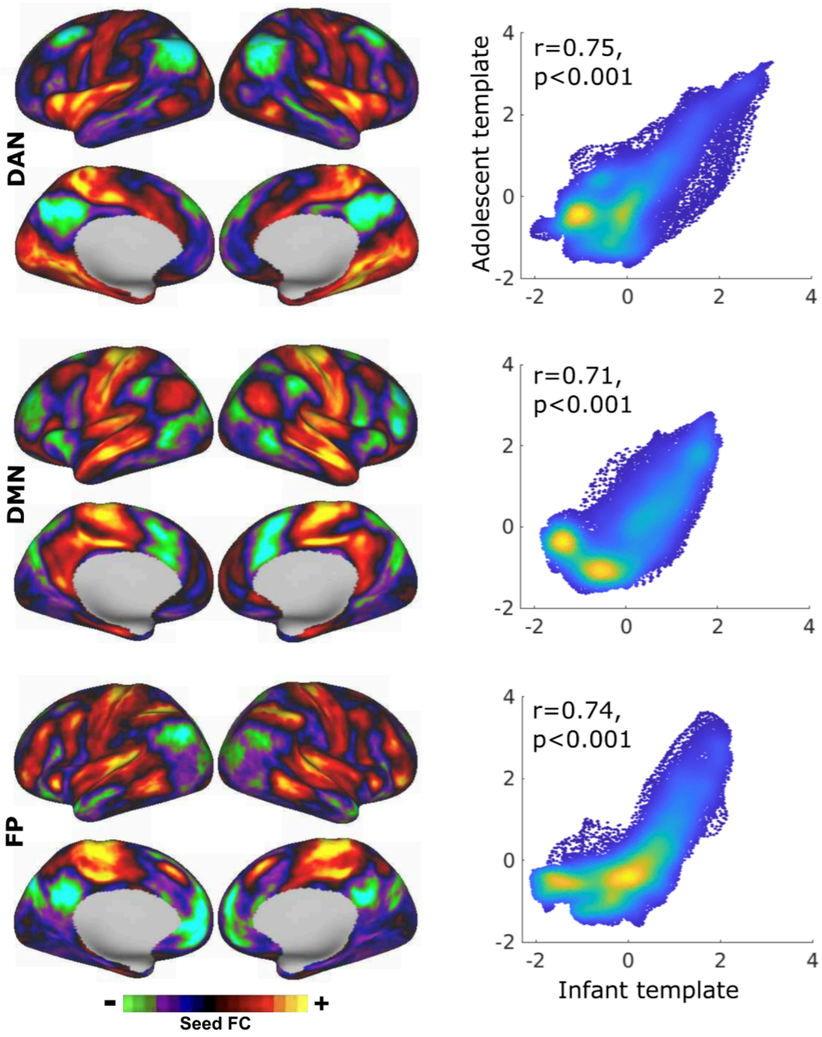

Supplementary Figure 3. Correlation between network templates for infant and adolescent templates for DAN, DMN and FP. To visualize the observations made in Figure 2 in an alternative way, we subtracted the infant from the adolescent templates to create the difference plots shown in the left column and correlated the non-zero value grayordinates between them for each template (scatter plots to right network difference pilot). The connectivity patterns of each template was highly correlated between infant and adolescent. However, the distribution of values, indicated by colors representing the relative data density estimate (warmer colors indicate higher density), reinforces the general observation made in Figure 2 that certain regions of the infant template are more negatively correlated to the seed region of a given network compared to the adolescent templates.

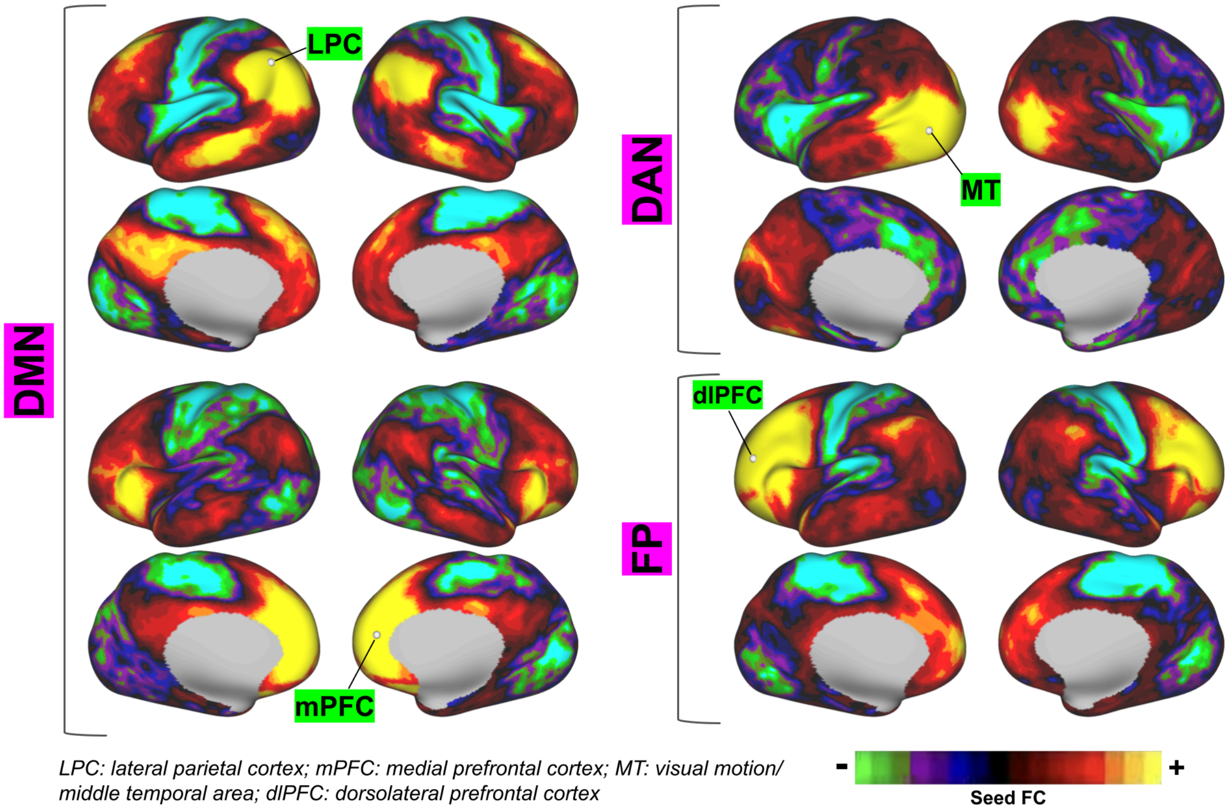

Supplementary Figure 4. Exemplary seed maps from the average dense connectivity matrix of the neonatal UCI cohort used to generate the set of infant network templates. To illustrate that the connectivity of the infant network templates is based on the resting state connectivity of the UCI infant cohort used to generate the templates (as opposed to an artifact from using the adult network definitions as priors when generating the templates), we placed seed regions (*green highlighted text*) in various regions of the brain to illustrate that these regions show similar patterns of connectivity compared to relevant infant network templates (*purple highlighted text*) displayed in Figure 2. The color bar displays the strength and valence of connectivity between a given seed region and the rest of the brain, with cooler and warmer colors representing negative and positive resting state connectivity respectively. Seeds placed in the lateral parietal cortex (**LPC**) and medial prefrontal cortex (**mPFC**) showed similar patterns of connectivity to the DMN template. The visual motion/middle temporal area (**MT**) and dorsolateral prefrontal cortex (**dlPFC**) displayed similar patterns of connectivity compared to the infant network templates for DAN and FP, respectively.

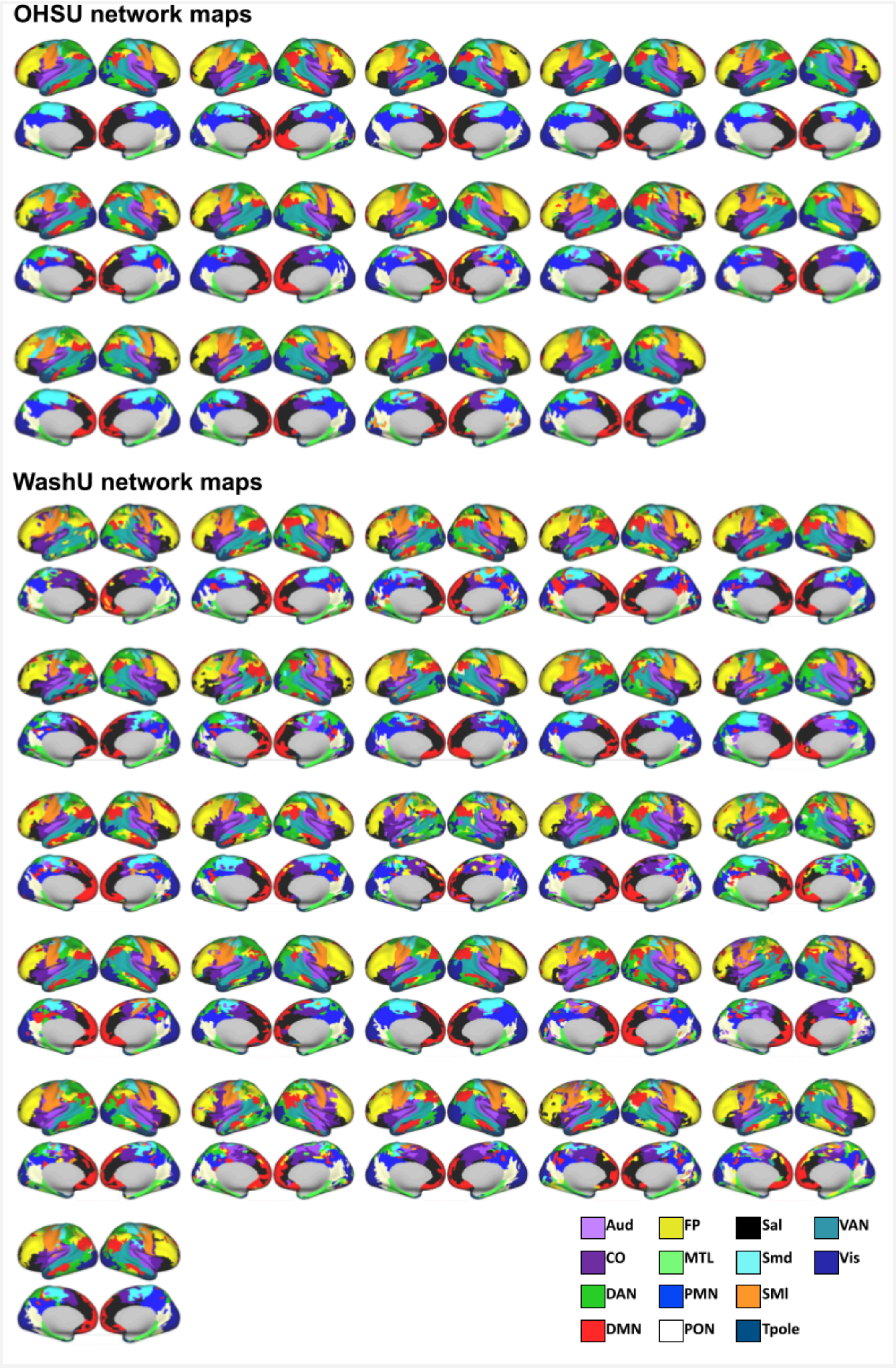

Supplementary Figure 5. Individualized network maps generated for OHSU and WashU subjects using template matching.

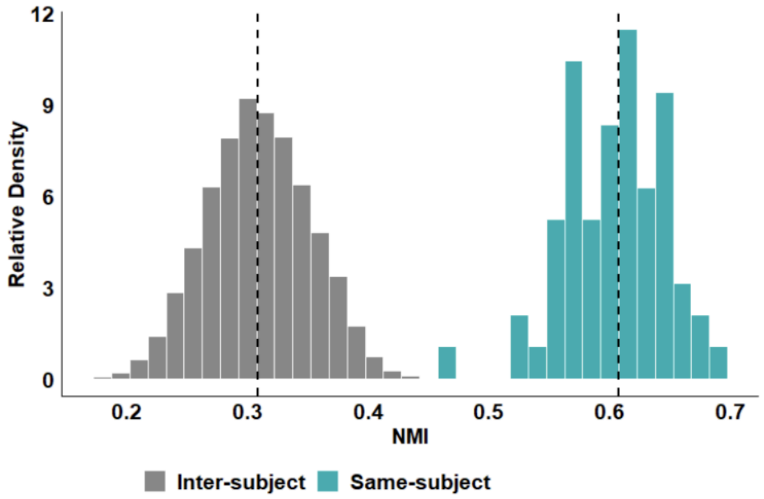

Supplementary Figure 6. Individualized network maps in UCI cohort derived from template matching based on infant versus adolescent network templates. In these histograms, same-subject (0.60±0.005) and inter-subject (0.31±0.0004) NMI is computed between adolescent- vs infant-based network maps derived from the full amount of resting state data. Same-subject NMI was significantly greater than inter-subject NMI (t=55.24, df=8126, p<0.0001, t-test), indicating that individualized network maps are agnostic to which set of templates is used. *******p<0.0001*

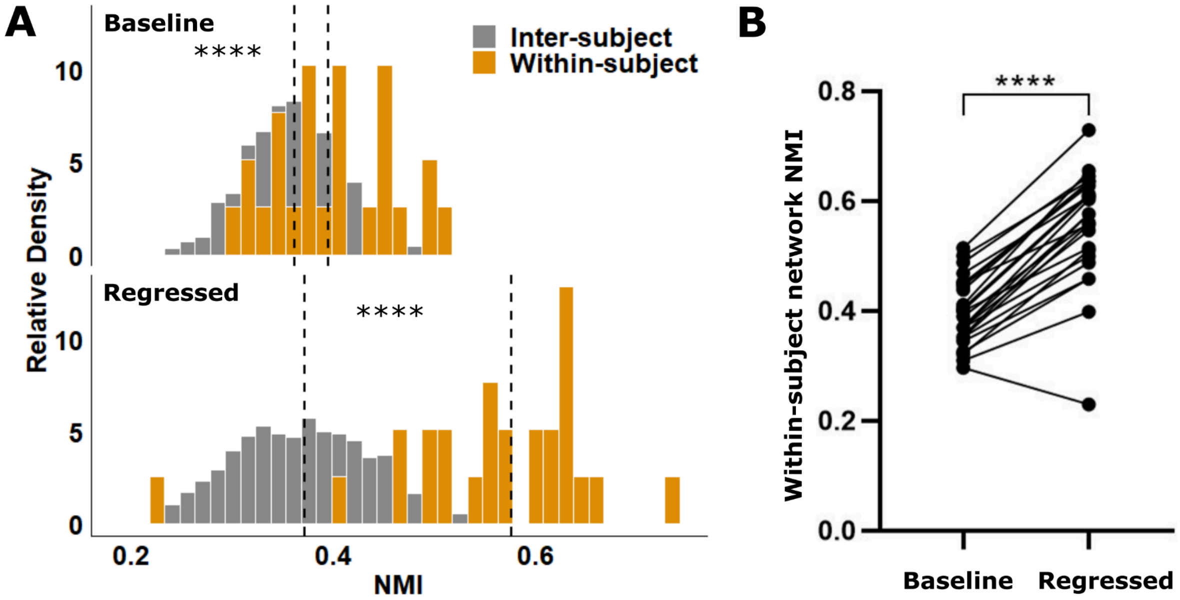

Supplementary Figure 7. Split-halves reliability analysis performed with baseline vs task-regressed WashU data. A: We generated network maps and repeated the split-halves reliability analysis in the WashU cohort data prior to regression of the auditory oddball response (upper histogram). As with the results derived from task-regressed data (lower histogram, copied from Figure 3A for visualization purposes), the resulting networks were individual-specific (within-subject NMI 0.40±0.01, inter-subject NMI: 0.36±0.00; t=3.953, df=1324, p<0.0001, t test). **B**: The main impact of task regression was that within-subject network similarity increased significantly by 0.16 NMI (full statistics in main text). There was also a small but significant increase in the similarity of inter-subject network maps when using regressed (0.37±0.002 NMI) versus auditory task data (0.36±0.001 NMI) (U=780697, p=0.0008, Mann-Whitney U). **** *p<0.0001*

Supplementary Methods

Volumetric Data Processing

The first stage of the default HCP pipeline, PreFreesurfer, takes a set of anatomical T1-weighted and T2-weighted raw images and computes a linear transform to a standard template, a brain mask, relevant image restoration, and a nonlinear registration to a standard template (Glasser et al., 2013). Several key modifications were made for adaptation to infant images. First, we applied ANTs DenoiseImage (Manjón et al., 2010) and N4BiasFieldCorrection (Tustison et al., 2010) in order to model Rician scanner noise and bias field inhomogeneities respectively. We then used a rigid body transform to ACPC-align both T1w and T2w structural images to the MNI neonate template from the NIH pediatric template (Fonov et al., 2009).

Brain extraction was performed by applying the T2w nonlinear warp from atlas-to-native space to the atlas brain mask. The brain mask is a version of the neonate template from the NIH pediatric atlas that we manually corrected to have tighter masking. The resulting T2w brain mask is converted to a T1w brain mask via rigid body registration of the T2w to T1w image and applying the resulting warp to the T2w brain mask. After T1w masking, the output of this process is the initial skull-stripped T1w and T2w infant brain in native volume space. We refine our extracted brains by computing tissue classes in the T2w brain using the ANTs Atropos (Avants et al., 2011) algorithm and applying the GM/WM masks to the T1w brain. The masked T1w and T2w are then nonlinearly registered to the MNI infant atlas. Next, the brain segmentation is further refined using a set of T1w infant atlases with labeled brain regions. UCI was processed using a set of 10 T1w anatomical images and their segmentations from ABCD. The OHSU and WashU cohorts were segmented using a set of 20 neonatal ALBERT T1w images[1] (Gousias et al., 2012, 2013) with corresponding segmentations and masks generated using the DCAN-infant pipeline followed by manual refinement. Each atlas is nonlinearly registered to the subject T1w, then the ANTS Joint Label Fusion (Wang & Yushkevich, 2013) algorithm is used to compute the local cross correlations between voxel intensities in the subject and each atlas. Each atlas then "votes" on how each voxel kernel in the subject brain should be labeled, based on how correlated the intensities between each voxel kernel in the subject and atlas are in a given brain region.

The second stage of the pipeline, Freesurfer, adjusts the mean intensity of white and grey matter labels to match the Freesurfer adult atlas, reconstructs native surfaces from the normalized structural data, and registers the surfaces to the MNI atlas surface in a point-to-point mapping. Next, PostFreeSurfer converts restored volumes native surfaces into HCP-compatible format (i.e. CIFTIs), and transforms the surfaces geometrically into atlas space. For the last step, our pipeline uses the ANTS

[1] Copyright Imperial College of Science, Technology and Medicine and Ioannis S. Gousias 2013. All rights reserved.

compressible fluid deformation algorithm to perform nonlinear registration instead of the FNIRT elastic deformation algorithm used in the HCP pipeline.

The last stage of volumetric data processing, fMRIVolume, registers the functional data to the standard template through the restored anatomical data. No distortion correction was performed for UCI subjects.

Surface Processing

After fMRIVolume, fMRISurface projects the functional data onto the template-space surfaces via nonlinear registration for cortical projection. In contrast to the HCP pipeline (Glasser et al., 2013), the infant-abcd-hcp-pipeline (Sturgeon et al., 2023) handles the subcortical projection slightly differently and registers subcortical ROIs to adult space by performing a linear registration of each subcortical ROI to adult space separately. In order to make our results more comparable to findings in adults as well as prior infant research that similarly uses only cortical data, we excluded subcortical data from the dense time series and generated network maps using only cerebral cortical data for the analyses performed in this paper.

The infant-ABCD-HCP-pipeline has 2 additional steps: 1) DCANBOLDproc for functional connectivity pre-processing and 2) ExecutiveSummary that creates standard outputs for quality control. DCANBOLDproc performs standard preprocessing on rest fMRI scans: first all fMRI data are demeaned and detrended with respect to time. Next a general linear model is used to denoise the processed fMRI data. Denoising regressors comprise signal and movement variables. Signal variables comprise mean time series and first derivative for white matter, CSF, and the global signal, which are derived from Individualized segmentations generated during PostFreesurfer. Movement variables comprise translational (X,Y,Z) and rotational (roll, pitch, and yaw) measures estimated by realignment during fMRIVolume and their Volterra expansion. These regressors represent the best approach to denoising currently studied. In particular, the inclusion of GSR is critical for most resting-state functional MRI comparisons, as demonstrated empirically by multiple independent labs. After denoising the fMRI data, the timeseries are bandpass filtered between 0.008 and 0.09 Hz using a 2nd order butterworth filter. Such a bandpass filter is softer than other filters, and avoids potential aliasing of the time series signal.

After standard preprocessing, motion censoring is performed using framewise displacement (FD), calculated as the squared sum of all the motion vectors estimated during frame-frame alignment. For standard preprocessing, betas for denoising are calculated using data with frames censored if they exceed a FD of 0.3 mm. For bandpass filtering, interpolation is used initially to replace the censored frames, and the residuals are extracted from the denoising GLM. In this way, standard preprocessing of the timeseries only uses data below the FD threshold, but avoids potential aliasing due to missing timepoints.

ExecutiveSummary, the final stage of the pipeline, produces a visual summary to enable visual inspection of outputs for quality control. This step is critical because, unlike volume-based pipelines, surface-based pipelines do not have metrics that enable simple quality control to exclude subjects. Any data that did not pass our standard operating procedure for assessing quality control were excluded from analysis.

References

Avants, B. B., Tustison, N. J., Wu, J., Cook, P. A., & Gee, J. C. (2011). An open source multivariate framework for n-tissue segmentation with evaluation on public data. *Neuroinformatics*, *9*(4), 381–400. https://doi.org/10.1007/s12021-011-9109-y

Fonov, V. S., Evans, A. C., McKinstry, R. C., Almli, C. R., & Collins, D. L. (2009). Unbiased nonlinear average age-appropriate brain templates from birth to adulthood. *NeuroImage*, *Supplement 1*(47), S102. https://doi.org/10.1016/S1053-8119(09)70884-5

Glasser, M. F., Sotiropoulos, S. N., Wilson, J. A., Coalson, T. S., Fischl, B., Andersson, J. L., Xu, J., Jbabdi, S., Webster, M., Polimeni, J. R., Van Essen, D. C., Jenkinson, M., & WU-Minn HCP Consortium. (2013). The minimal preprocessing pipelines for the Human Connectome Project. *NeuroImage*, *80*, 105–124. https://doi.org/10.1016/j.neuroimage.2013.04.127

Gousias, I. S., Edwards, A. D., Rutherford, M. A., Counsell, S. J., Hajnal, J. V., Rueckert, D., & Hammers, A. (2012). Magnetic resonance imaging of the newborn brain: manual segmentation of labelled atlases in term-born and preterm infants. *NeuroImage*, *62*(3), 1499–1509. https://doi.org/10.1016/j.neuroimage.2012.05.083

Gousias, I. S., Hammers, A., Counsell, S. J., Srinivasan, L., Rutherford, M. A., Heckemann, R. A., Hajnal, J. V., Rueckert, D., & Edwards, A. D. (2013). Magnetic resonance imaging of the newborn brain: automatic segmentation of brain images into 50 anatomical regions. *PloS One*, *8*(4), e59990. https://doi.org/10.1371/journal.pone.0059990

Manjón, J. V., Coupé, P., Martí-Bonmatí, L., Louis Collins, D., & Robles, M. (2010). Adaptive non-local means denoising of MR images with spatially varying noise levels. In *Journal of Magnetic Resonance Imaging* (Vol. 31, Issue 1, pp. 192–203). https://doi.org/10.1002/jmri.22003

Sturgeon, D., Snider, K., Moore, L.A., Perrone, A.J., Earl, E., Madison, T.J., Conan, G., Klein, R., Miranda-Dominguez, O., Feczko, E., Graham, A.M., Fair, D.A., 2023. DCAN-Labs infant-abcd-bids-pipeline (v0.0.22) [WWW Document]. doi:10.5281/zenodo.7683282

Tustison, N. J., Avants, B. B., Cook, P. A., Zheng, Y., Egan, A., Yushkevich, P. A., & Gee, J. C. (2010). N4ITK: improved N3 bias correction. *IEEE Transactions on Medical Imaging*, *29*(6), 1310–1320. https://doi.org/10.1109/TMI.2010.2046908

Wang, H., & Yushkevich, P. (2013). Multi-atlas segmentation with joint label fusion and corrective learning—an open source implementation. *Frontiers in Neuroinformatics*, *7*, 27. https://doi.org/10.3389/fninf.2013.00027
